# Machine-learning-based prediction of a diagnostic model using autophagy-related genes based on RNA sequencing for patients with papillary thyroid carcinoma

**DOI:** 10.1515/med-2024-0896

**Published:** 2024-02-05

**Authors:** Lin Chen, Gaofeng Tao, Mei Yang

**Affiliations:** Department of Endocrinology and Metabolism, People’s Hospital of Chongqing Liang jiang New Area, Chongqing, China; Department of Medicine and Education, People’s Hospital of Chongqing Liang jiang New Area, Chongqing, China

**Keywords:** papillary thyroid carcinoma, immune microenvironment, diagnosis, machine learning, biomarker, autophagy, RNA sequencing

## Abstract

Papillary thyroid carcinoma (PTC) is the most common type of thyroid cancer and belongs to the category of malignant tumors of the thyroid gland. Autophagy plays an important role in PTC. The purpose of this study is to develop a novel diagnostic model using autophagy-related genes (ARGs) in patients. In this study, RNA sequencing data of PTC samples and normal samples were obtained from GSE33630 and GSE29265. Then, we analyzed GSE33630 datasets and identified 127 DE-ARGs. Functional enrichment analysis suggested that 127 DE-ARGs were mainly enriched in pathways in cancer, protein processing in endoplasmic reticulum, toll-like receptor pathway, MAPK pathway, apoptosis, neurotrophin signaling pathway, and regulation of autophagy. Subsequently, CALCOCO2, DAPK1, and RAC1 among the 127 DE-ARGs were identified as diagnostic genes by support vector machine recursive feature elimination and least absolute shrinkage and selection operator algorithms. Then, we developed a novel diagnostic model using CALCOCO2, DAPK1, and RAC1 and its diagnostic value was confirmed in GSE29265 and our cohorts. Importantly, CALCOCO2 may be a critical regulator involved in immune microenvironment because its expression was related to many types of immune cells. Overall, we developed a novel diagnostic model using CALCOCO2, DAPK1, and RAC1 which can be used as diagnostic markers of PTC.

## 1 Introduction

One of the most rapidly developing tumors worldwide, thyroid carcinoma (TC) is also the most common endocrine malignancy [1]. Well-differentiated papillary thyroid carcinoma (PTC) is the most common kind of thyroid carcinoma. There are five subtypes based on histological criteria [2]. PTC is more commonly observed in females than in males, with a higher incidence rate among women. This gender disparity may be attributed to hormonal levels and other biological differences [3]. Even though PTC is a global problem, regional variations in prevalence are possible. Incidence rates of the illness may vary from region to region depending on a number of factors, including geography, climate, and family history [4,5]. Exposure to certain forms of radiation is linked to an elevated risk of thyroid cancer, particularly instances such as childhood exposure to radioactive iodine treatments and exposure to radioactive contamination resulting from nuclear accidents [6]. However, these circumstances are typically more closely related to other types of thyroid cancer rather than solely to PTC. Some patients with PTC may develop more aggressive illness, such as nerve and vascular invasion, local and distant metastasis, and recurrence, which will less react satisfactorily to standard therapy, eventually resulting to poor patient survival despite the high prognosis [7,8]. Early diagnosis plays a pivotal role in managing PTC. It holds the promise of enhancing patient prognosis, minimizing the invasiveness of therapy, reducing the risk of disease spread and metastasis, expanding treatment options for physicians, and positively impacting patients’ mental and emotional well-being. Regular check-ups, coupled with diagnostic methods like thyroid ultrasound, contribute significantly to the early detection of PTC and other thyroid disorders, thereby leading to enhanced treatment outcomes and overall quality of life [9]. Thus, new molecular pathogenic indicators and therapeutic targets are needed for early detection and effective treatment.

Autophagy is a crucial intracellular biological process (BP) through which cells degrade and eliminate their own harmful or damaged components, maintaining cellular homeostasis and functionality [10]. Regarded as an adaptive response, autophagy is initiated when cells face conditions such as nutrient scarcity, oxygen deficiency, protein aggregation, infection, or other stressors, providing the cell with necessary energy and materials [11,12,13]. The fundamental process of autophagy involves three key steps: First, a double-membrane vesicle called an autophagosome forms around damaged or aging organelles, protein aggregates, and other cellular components (CC). Next, the autophagosome fuses with lysosomes within the cell, creating an autolysosome. Lastly, enzymes within the autolysosome degrade the enclosed components, releasing amino acids, lipids, and other useful molecules for cellular use [14]. Autophagy plays a vital role in maintaining cellular internal environment balance, waste disposal, metabolic regulation, and protein quality control [15]. However, it also plays a role in the pathogenesis of various diseases, including neurodegenerative diseases, inflammatory conditions, and tumors. Within tumors, the role of autophagy is complex. On one hand, by increasing cell apoptosis (programmed cell death), autophagy can decrease tumors and slow their progression [16,17]. Moreover, autophagy can play a role in eliminating potential harmful factors that initiate tumor formation, including DNA damage and abnormal proteins, thereby decreasing the probability of cell transformation into cancer cells. Conversely, some studies propose that specific tumor cells may leverage autophagy to adapt to challenging conditions such as low oxygen and nutrient deprivation. This adaptation allows tumor cells to endure stress and potentially results in the emergence of treatment resistance [18,19]. Consequently, autophagy is sometimes considered a mechanism in tumors that counters therapeutic interventions. Overall, the role of autophagy in tumor development depends on the tumor type, environmental factors, and specific molecular regulatory mechanisms within cells. Researchers are diligently investigating this process to develop more precise therapeutic strategies, which might involve intervening in the autophagy process to control or guide tumor growth.

Machine learning is a subfield of artificial intelligence that aims to enable computer systems to learn from experience using data and statistical techniques, improve their performance, and gradually enhance their ability to perform tasks without requiring explicit programming [20]. The goal of machine learning is to develop algorithms and models that allow computers to learn and optimize automatically based on data [21]. This learning process involves identifying patterns from data, generating predictive models, and making accurate predictions or decisions when confronted with new data [22]. Machine learning plays a crucial role in the medical field, particularly in tumor diagnosis and treatment, by predicting tumor biomarkers. Tumor biomarkers are certain molecules within the body whose presence and changes might be associated with tumor development, progression, and treatment response [23,24]. The application of machine learning encompasses several aspects: First, through analyzing medical images, patient information, and tumor biomarker data, machine learning assists doctors in early and accurate identification of whether a patient has a tumor. Second, by analyzing time-series data of tumor biomarkers, machine learning can predict tumor growth rates and spread trends, supporting the creation of personalized treatment plans and monitoring treatment effectiveness [25,26]. Furthermore, based on machine learning models, it can forecast how different treatment methods will affect a patient, aiding doctors in selecting the optimal treatment strategy and enhancing treatment success rates [27]. Simultaneously, by analyzing genetic information, tumor biomarkers, and health data, machine learning helps in tailoring individualized treatment plans for each patient, improving treatment efficacy and reducing side effects. Lastly, in drug development, machine learning can predict how drugs will interact with specific tumor biomarkers, accelerating the drug screening and development process [28]. In summary, the application of machine learning in predicting tumor biomarkers provides substantial support for tumor diagnosis, treatment, and drug development within the medical field. In recent years, more and more studies have used Machine learning and High-throughput Sequencing to screen diagnostic genes for PTC.

The tumor immune microenvironment (TME) refers to the complex environment consisting of various immune cells, blood vessels, extracellular matrix (ECM), and molecular signals present within tumor tissue [29]. The microenvironment is pivotal in shaping the formation, growth, progression, and response to the treatment of tumors. The composition and features of the TME exert a substantial influence on the biological behavior of tumors and the effectiveness of therapeutic interventions [30]. The primary components and roles of the TME are as follows: first, in addition to T cells and B cells, the TME also contains macrophages, dendritic cells, and other immune cells. They have immunological responses to tumor cells and interact with them. It is possible that tumor cells can control certain immune cells to aid tumor development while other immune cells have anti-tumor properties and actively seek out and destroy tumor cells. Second, immune cells within TME are capable of recognizing tumor-specific antigens, triggering immune cell attacks. However, tumor cells often evade immune system attacks by activating immune checkpoint pathways, thereby weakening immune responses [31,32]. The ECM constitutes a three-dimensional network structure crucial for supporting cell survival and interactions. Tumors have the capability to alter the ECM, influencing cell migration, infiltration, and signal transmission. Within TME, the vascular system plays a vital role in tumor development, as tumors necessitate sufficient oxygen and nutrients for growth. However, blood vessel structures in TME are often abnormal, potentially leading to inadequate oxygen supply within tumor tissues and affecting the function of immune cells. Lastly, Different cytokines and growth factors in the TME coordinate the activities of tumor cells, immune cells, and other cell types [33,34]. Overall, the TME plays a pivotal role in the development of tumors, impacting tumor cell behavior and the effectiveness of immunotherapies. Understanding and manipulating the TME is a critical focus of current tumor research and treatment endeavors. However, the potential mechanisms involved in genes regulating TME remained largely unclear.

In this study, we aimed to use Machine learning to screen diagnostic genes based on the autophagy-related genes (ARGs) for PTC patients and developed a novel diagnostic model for PTC. In addition, to provide the groundwork for future studies, we also investigated the connection between the detected biomarkers and invading immune cells.

## 2 Materials and methods

### 2.1 Clinical samples

A total of 12 paired PTC tissues and the matched adjacent normal tissues were obtained during surgery at People’s Hospital of Chongqing Liang Jiang New Area, between March 2022 and December 2022. Clinical and pathological findings were used to make a diagnosis, and specimens were snap-frozen and kept in liquid nitrogen chambers shortly after collection. Prior patient consents and approval from the Institutional Research Ethics Committee of People’s Hospital of Chongqing Liang Jiang New Area were acquired for the use of these clinical materials for research purposes.

### 2.2 RNA extraction and quantitative reverse transcription-PCR assays

Following the protocol provided by the manufacturer, total RNA was isolated from tissues or cultured cells using TRIzol reagent (Invitrogen). A PrimeScript RT Reagent Kit (TaKara, Dalian, China) was used to convert 1 μg of RNA into cDNA in a final volume of 20 μL under normal conditions. SYBR Premix Ex Taq (Takara) was used in an Applied Biosystems 7500 Real-Time PCR System for the PCR analysis. The relative threshold cycle approach was used to convert the expression levels of diagnostic genes to fold changes relative to GAPDH. Our qRT-PCR data were interpreted and reported as fold changes with respect to threshold cycle (CT) values.

### 2.3 Data extraction

Data on PTC gene expression patterns were initially culled from the GEO database maintained by the National Center for Biotechnology Information. GSE33630 (GPL570, 45 normal specimens and 49 PTC specimens) [35] and GSE29265 (GPL570, 20 normal specimens and 20 PTC specimens) were chosen as the two datasets to analyze based on the aforementioned criteria. MSigDB was utilized to compile the list of autophagy-associated genes (*n* = 178) used in this investigation.

### 2.4 Differential expression analysis

We began by retrieving expression data for 178 FRGs from the GSE33630 database, which included both normal and PTC samples. Next, we utilized the “limma” package in R to identify the ARGs that were expressed differently across the two samples (DE-ARGs). Significant genes have a *P*-value of 0.05 or below.

### 2.5 Functional enrichment analysis

Enrichment studies were performed using the “cluster profiler” R package, and ARG functions were predicted using the Gene Ontology (GO) function and the KEGG pathway. Molecular functions, BP, and CC are the three main divisions of the GO database. Information on chemical, genetic, and systemic functions is all available in the KEGG database. To identify significantly enriched GO keywords and KEGG pathways, we set the threshold at *P* < 0.05.

### 2.6 Screening of candidate diagnostic biomarkers

In this investigation, we employed the least absolute shrinkage and selection operator (LASSO) and the support vector machine (SVM) recursive feature elimination (SVM-RFE) to identify potentially important prognostic factors. SVM-RFE is a feature selection method used in machine learning to choose the most relevant and valuable subset of features, aiming to enhance model performance and generalization capability. It combines the concepts of the SVM classifier and the Recursive Feature Elimination algorithm. The advantages of SVM-RFE lie in its capability to efficiently reduce the number of features, enhance model interpretability, and, in situations where training data includes noise or redundant features, improve the model’s generalization performance. The regression that is applied using the “e1071” package. SVM-RFE was used to find genes with better discriminatory ability. LASSO is a statistical method used for feature selection and regularization, commonly applied in linear regression and machine learning models. It constrains the coefficients of the model, encouraging some features’ coefficients to approach zero, thereby achieving feature selection and model simplification to prevent overfitting. The advantage of LASSO lies in its ability to effectively handle high-dimensional data and automatically perform feature selection when dealing with a large number of features. By shrinking the coefficients of unimportant features towards zero, LASSO helps the model generalize better to new data while also providing interpretability for the various features within the model. LASSO was performed using the “glmnet” package in R. We used the GSE33630 dataset to test two methods, and the resulting overlap in genes was used to verify the expression levels of potential diagnostic biomarkers. In addition, To categorize samples in the GSE33630 and GSE29265 datasets, we constructed a logistic regression model with seven marker genes using the predict function in the R package glm. ROC curves were also used to assess the diagnostic efficacy of the logistic regression model.

### 2.7 Single-gene gene set enrichment analysis (GSEA) enrichment analysis

GSEA, a bioinformatics method, is employed for analyzing gene expression data to identify the enrichment level of functional features or pathways linked to a set of genes under specific biological conditions. Particularly well-suited for high-throughput gene expression data, such as microarray or RNA sequencing data, this approach aids in unraveling the intricate relationships within biological systems. The advantage of GSEA lies in its consideration not only of individual gene differential expression but also the expression patterns of entire gene sets, thus providing a more comprehensive perspective for functional analysis. It can uncover which functional pathways or features are associated with gene expression changes under different biological conditions, aiding in the interpretation of biological phenomena and offering new hypotheses and insights. This study is carried out using the GSEA (V.4.1.0) tool that is available in R. We analyzed the association between the seven marker genes and every other gene in the GSE33630 dataset to learn more about the pathways to which they belong. Meanwhile, KEGG signaling pathways were also analyzed for enrichment in the gene set by invoking them as a predetermined set.

### 2.8 Single-gene gene set variation analysis (GSVA) enrichment analysis

GSVA is a method used for analyzing gene expression data, aiming to reveal the variation of functional gene sets associated with a set of genes across different samples or conditions. Unlike traditional differential expression analysis that focuses on changes in individual genes, GSVA treats gene sets as units and considers the overall variation in gene expression. The R package known as GSVA (version 1.38.0.0) was used to carry out this research. The advantage of GSVA lies in its ability to comprehensively capture the changes in functional gene sets, thereby providing more accurate biological explanations. Compared to individual gene differential analysis, GSVA is better equipped to identify differences between samples or conditions by considering the entire gene set. This is highly valuable for understanding the overall changes in gene function and their association with BP. In this study, each marker gene underwent GSVA analysis, with the KEGG pathway set serving as the background gene set. Concurrently, we used the limma program to compare samples from high- and low-expression groups of the marker gene using the GSVA score difference.

### 2.9 Immune infiltration analysis

CIBERSORT is a computational method used to analyze gene expression data, aiming to estimate the relative abundance of different cell types within complex mixed cell samples [36]. This approach is applicable to gene expression data obtained from biological samples such as tissues or blood. The fundamental principle of CIBERSORT involves constructing a reference gene expression pattern database that includes gene expression features of various pure cell types. Then, the gene expression data from the mixed cell sample under analysis are compared to these reference patterns. CIBERSORT employs a linear model to infer the relative proportions of different cell types present within the mixed sample. This methodology helps decipher the contributions of distinct cell types within complex tissues or samples. Expression matrix-based CIBERSORT calculations were utilized to determine PTC patient-to-healthy person immune cell ratios, and the R package “vioplot” was used to graphically represent these ratios for 22 different immune cell types. The quantitative association between various immune cells was shown as a heat map using the “corrplot” software. Additionally, the “ggplot2” R software was used to analyze the correlation between diagnostic marker expression and immune cell ratios.

### 2.10 Statistical analysis

We performed the statistical analyses using R (version 4.1.1). Continuous variables were expressed as median (interquartile range) and compared using the Mann–Whitney *U* test, while categorical variables are expressed as frequencies (*n* [%]) and compared using the *χ* ^2^ or Fisher exact test, when appropriate. ROC analysis was applied to examine the diagnostic value of the new model for PTC. A *P*-value of less than 0.05 was considered statistically significant.

## 3 Results

### 3.1 Identification of DE-ARGs in PTC specimens and functional enrichment analysis

**Figure 1:**
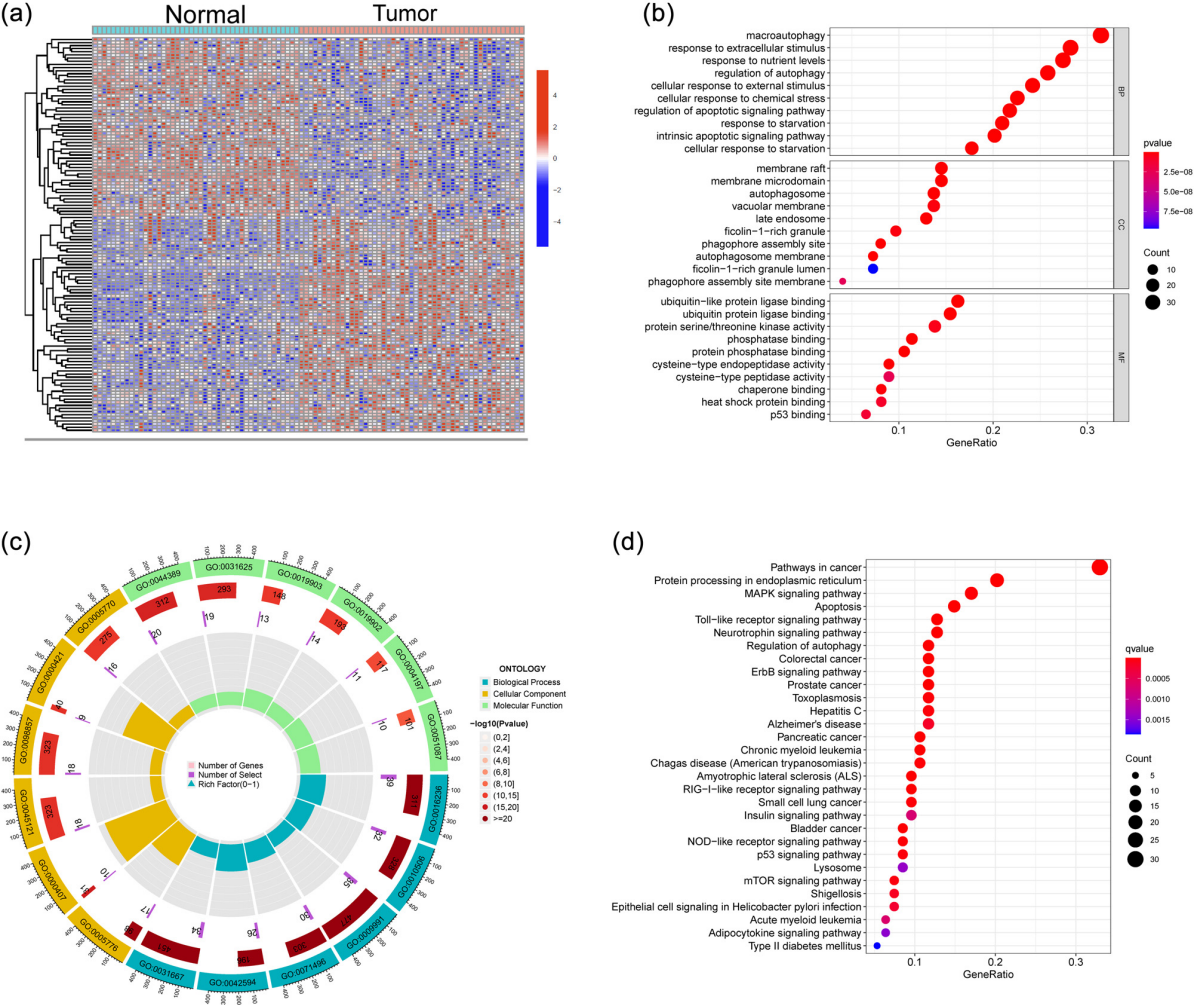
Identification of DE-ARGs in PTC specimens from GSE33630 datasets. (a) Heatmap of DE-ARGs in PTC specimens. (b and c) GO enrichment analysis. (d) KEGG pathway enrichment analysis.

Data from a total of 49 PTC and 45 normal samples from GSE33630 datasets were retrospectively analyzed. The DE-ARGs were analyzed using the limma package. A total of 127 DE-ARGs were obtained: 69 genes were significantly upregulated and 57 genes were significantly downregulated (Figure 1a). To explore the potential function of DE-ARGs, we performed GO assays and the results indicated that 127 DE-ARGs were mainly enriched in macroautophagy, response to extracellular stimulus, response to nutrient levels, regulation of autophagy, membrane raft, membrane microdomain, autophagosome, vacuolar membrane, ubiquitin-like protein ligase binding, ubiquitin protein ligase binding, phosphatase binding, and protein serine/threonine kinase activity (Figure 1b and c). Moreover, the results of KEGG assays indicated that 127 DE-ARGs were mainly associated with Pathways in cancer, Protein processing in endoplasmic reticulum, MAPK signaling pathway, apoptosis, toll-like receptor signaling pathway, neurotrophin signaling pathway, and regulation of autophagy (Figure 1d).

### 3.2 Three DE-ARGs were identified as diagnostic genes for PTC

**Figure 2:**
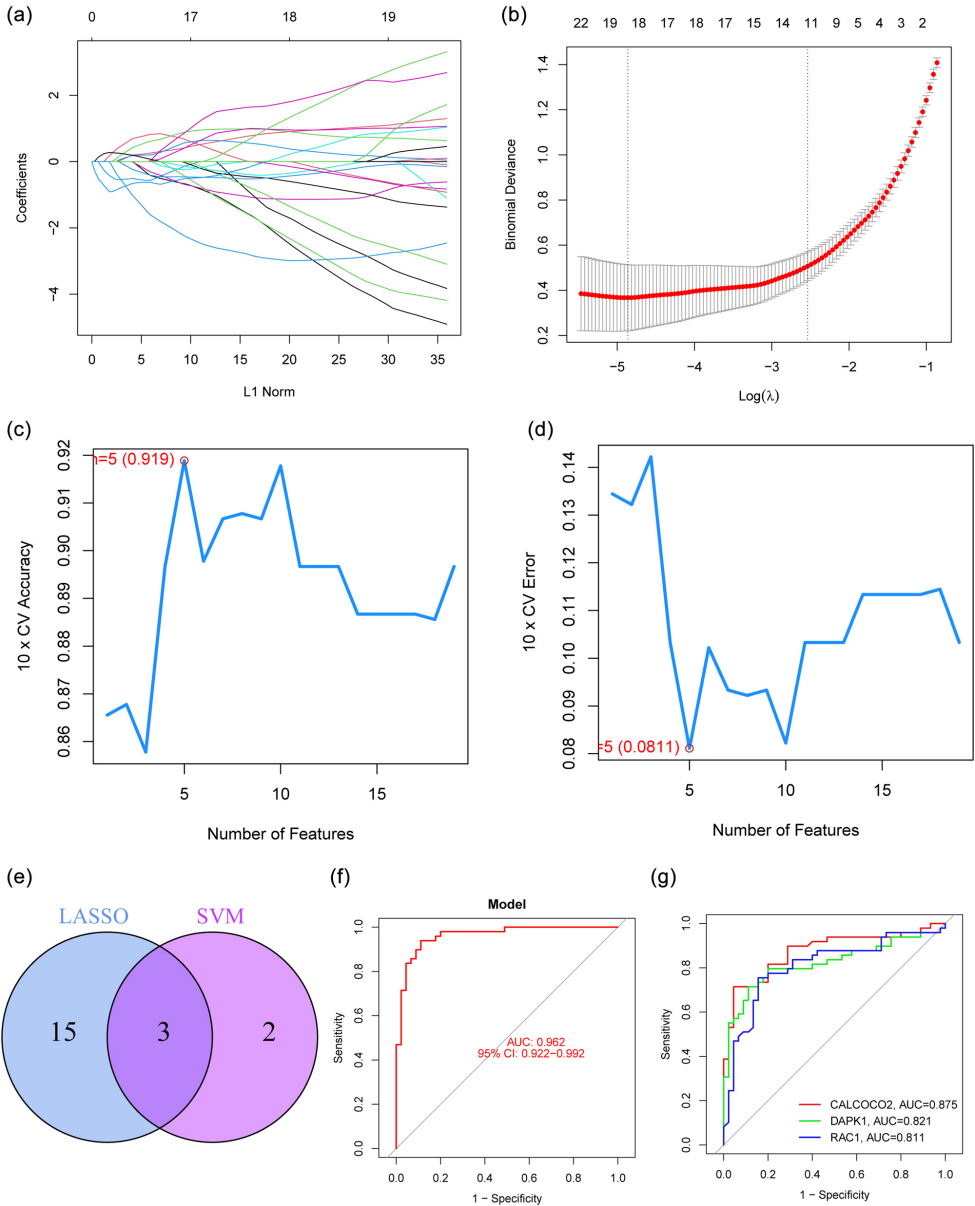
Identification of diagnostic biomarkers. (a and b) Cross-validation is employed to fine-tune parameter selection in LASSO regression. This approach involves partitioning the dataset into multiple subsets, with one subset used for validation while the others are used for training the model. Through iterative experimentation with different parameter values, the optimal configuration can be determined, thereby minimizing overfitting and maximizing predictive accuracy of the LASSO regression model. (c and d) Performing LASSO regression on a set of 13 module genes. This involves analyzing the relationships among these genes to identify the most influential ones. (e) Using a Venn diagram, we were able to identify genes from the two different machine learning strategies that cross. (f) Logistic regression model to identify the AUC of disease samples. (g) ROC curves for the three marker genes.

**Figure 3:**
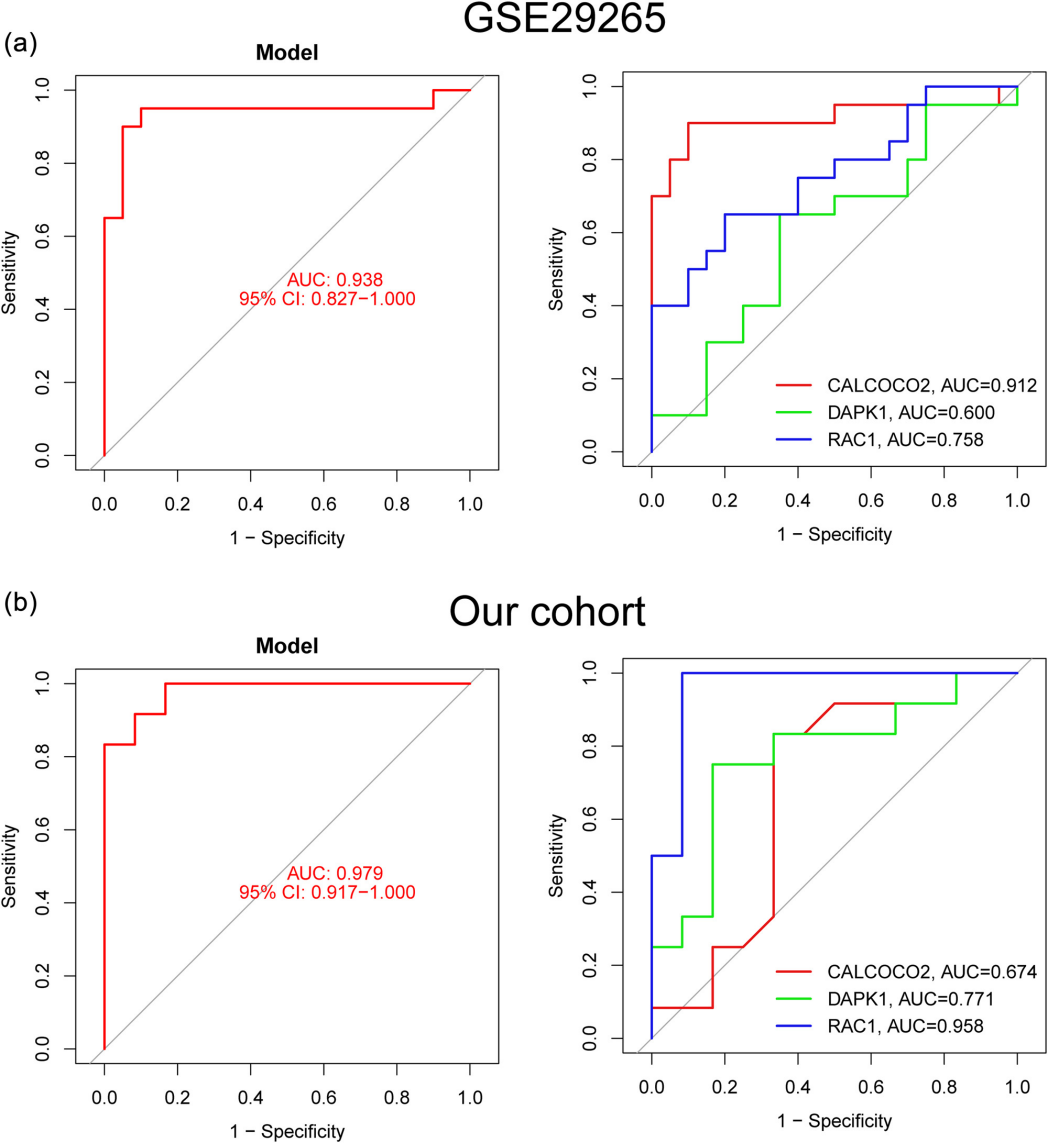
The diagnostic value of the new model was confirmed in (a) GSE29265 datasets and (b) our cohort.

**Figure 4:**
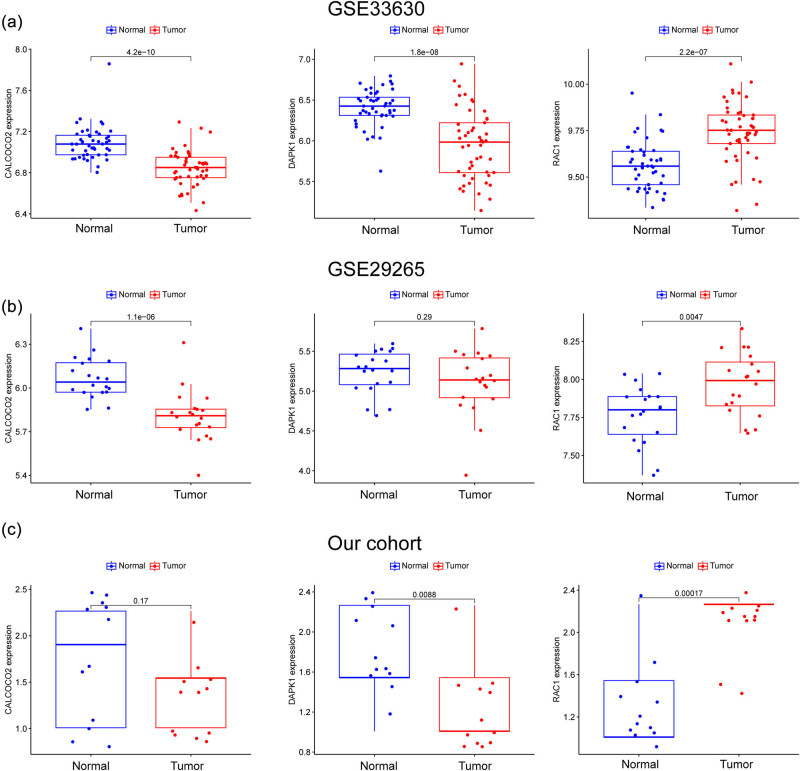
The expressing pattern of CALCOCO2, DAPK1, and RAC1 in (a) GSE33630 datasets, (b) GSE29265 datasets, and (c) our cohorts.

By comparing PTC patients to healthy controls, we hoped to gauge the diagnostic utility of DE-ARGs. We next used the LASSO and SVM-RFE machine learning algorithms on the GSE33630 dataset to separate PTC samples from control samples. In order to pick 18 PTC-related characteristics, the LASSO logistic regression technique was utilized, and the penalty parameter tuning process was carried out using 10-fold cross-validation (Figure 2a and b). Next, we used the SVM-RFE technique to narrow down the DE-FRGs and zero in on the best candidate feature gene sets. In the end, the five best candidate feature genes were selected (Figure 2c and d). Through an intersection of the LASSO and SVM-RFE models’ marker genes, we were able to isolate three candidate genes (CALCOCO2, DAPK1, and RAC1) for further study (Figure 2e). The logistic regression model that was based on these three marker genes was able to successfully differentiate between normal and PTC samples with an area under the curve (AUC) of 0.962, according to the results of our ROC curve analyses (Figure 2f). Moreover, ROC curves were created for the three marker genes to highlight their efficacy in discriminating PTC from normal samples. Figure 2g demonstrates that the AUC was more than 0.8 for all genes. Moreover, the diagnostic value of the new model was further confirmed in GSE29265 datasets (Figure 3a) and our cohorts (Figure 3b). Finally, the expression pattern of CALCOCO2, DAPK1, and RAC1 in different datasets is shown in Figure 4a–c. Importantly, we found that the expression of CALCOCO2 and DAPK1 was distinctly decreased in PTC specimens, while the expression of RAC1 was distinctly increased in PTC specimens. In light of what has been discussed thus far, it would appear that the logistic regression model is superior to the individual marker genes in terms of its accuracy and specificity when it comes to differentiating PTC samples from normal ones.

### 3.3 GSEA and GSVA enrichment analysis of three marker genes

**Figure 5:**
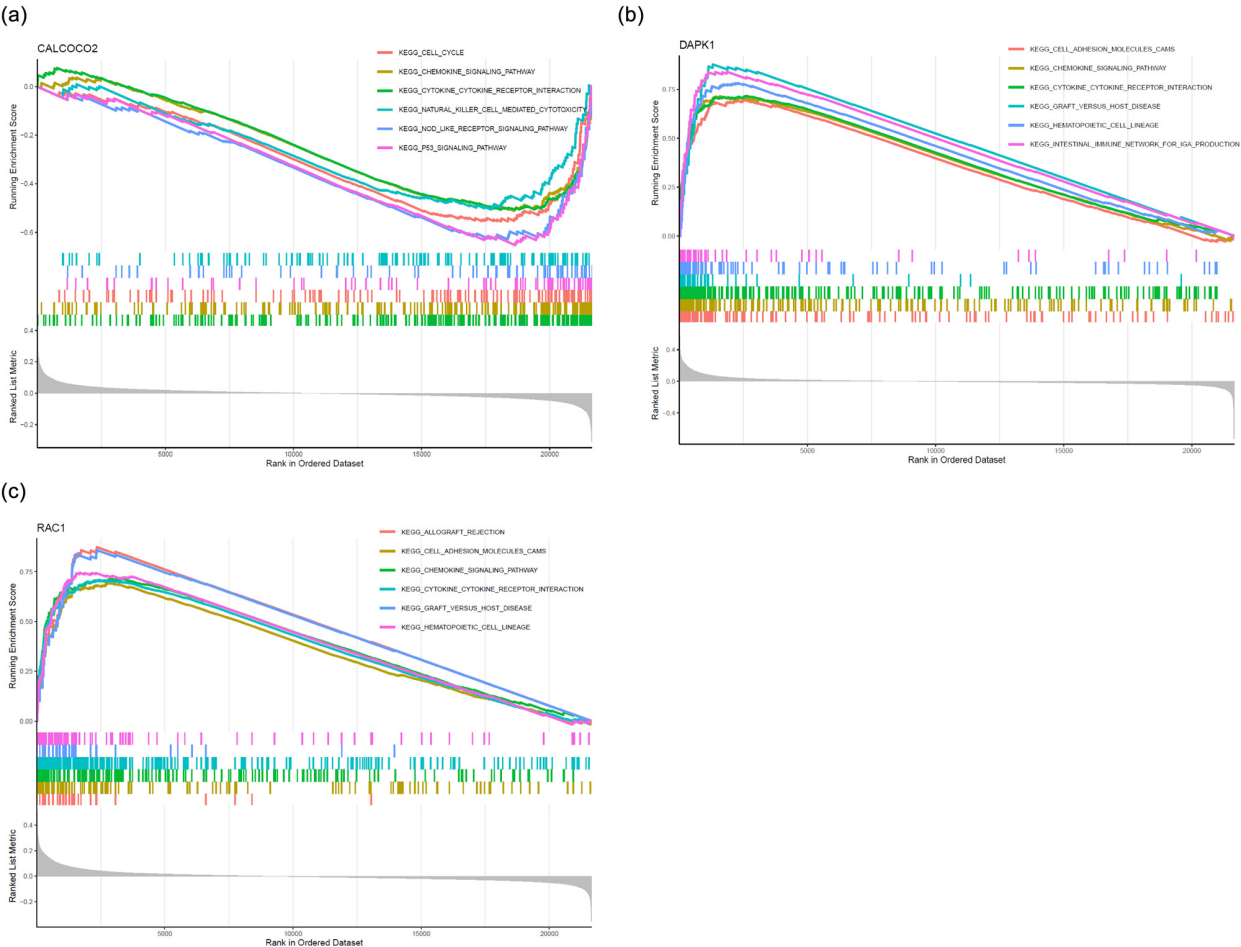
Single-gene GSEA-KEGG pathway analysis in (a) CALCOCO2, (b) DAPK1, and (c) RAC1.

**Figure 6:**
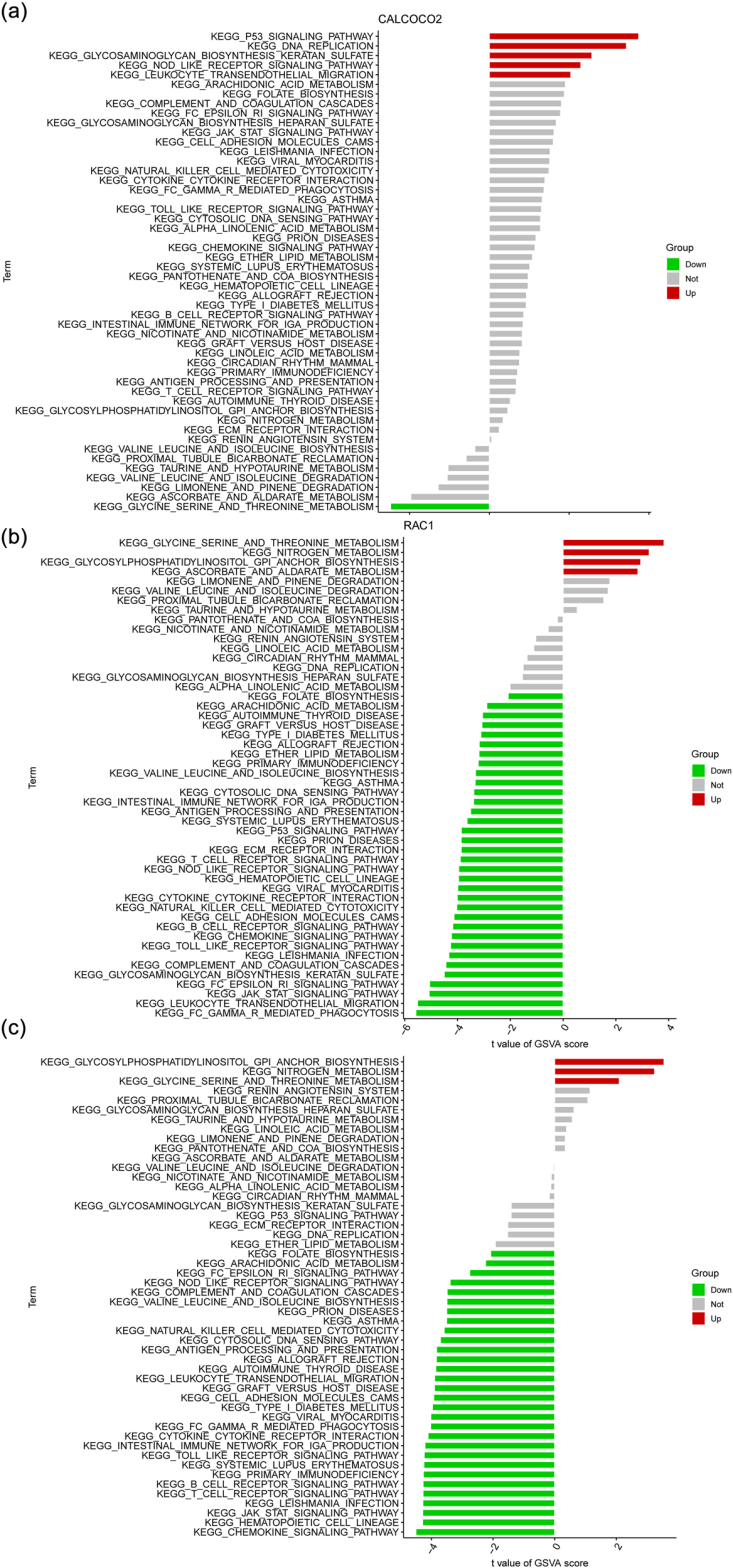
On the basis of the levels of expression of each marker gene in combination with GSVA, high- and low-expression groups were identified in (a) CALCOCO2, (b) DAPK1, and (c) RAC1.

We performed a gene-specific Gene Set Enrichment study-KEGG pathway study to learn more about the role that marker genes may have in separating healthy and sick samples. The genes in CALCOCO2 low-expression group were mainly enriched in CELL_CYCLE, CHEMOKINE_SIGNALING_PATHWAY, CYTOKINE_CYTOKINE_RECEPTOR_INTERACTION, NATURAL_KILLER_CELL_MEDIATED_CYTOTOXICITY, NOD_LIKE_RECEPTOR_SIGNALING_PATHWAY, and P53_SIGNALING_PATHWAY (Figure 5a). The genes in DAPK1 high-expression group were mainly enriched in CELL_ADHESION_MOLECULES_CAMS, CHEMOKINE_SIGNALING_PATHWAY, YTOKINE_CYTOKINE_RECEPTOR_INTERACTION, GRAFT_VERSUS_HOST_DISEASE, HEMATOPOIETIC_CELL_LINEAGE, and INTESTINAL_IMMUNE_NETWORK_FOR_IGA_PRODUCTION (Figure 5b). Moreover, the genes in RAC1 high-expression group were mainly enriched in ALLOGRAFT_REJECTION, CELL_ADHESION_MOLECULES_CAMS, CHEMOKINE_SIGNALING_PATHWAY, CYTOKINE_CYTOKINE_RECEPTOR_INTERACTION, GRAFT_VERSUS_HOST_DISEASE, and HEMATOPOIETIC_CELL_LINEAGE (Figure 5c). Then, we detected the pathways that were activated differently between the high-expression and low-expression groups by comparing the levels of expression of each marker gene in conjunction with GSVA. The results showed that the up-regulation of CALCOCO2 activated the P53_SIGNALING_PATHWAY, DNA_REPLICATION, GLYCOSAMINOGLYCAN_BIOSYNTHESIS_KERATAN_SULFATE, NOD_LIKE_RECEPTOR_SIGNALING_PATHWAY, and EUKOCYTE_TRANSENDOTHELIAL_MIGRATION (Figure 6a). In addition, the down-regulation of DAPK1 may activate CHEMOKINE_SIGNALING_PATHWAY, HEMATOPOIETIC_CELL_LINEAGE, JAK_STAT_SIGNALING_PATHWAY, and LEISHMANIA_INFECTION (Figure 6b). Finally, we observed that the down-regulation of RAC1 may activate FC_GAMMA_R_MEDIATED_PHAGOCYTOSIS, LEUKOCYTE_TRANSENDOTHELIAL_MIGRATION, JAK_STAT_SIGNALING_PATHWAY, and FC_EPSILON_RI_SIGNALING_PATHWAY (Figure 6c).

### 3.4 Correlation analysis between the three biomarkers and infiltrating immune cells

**Figure 7:**
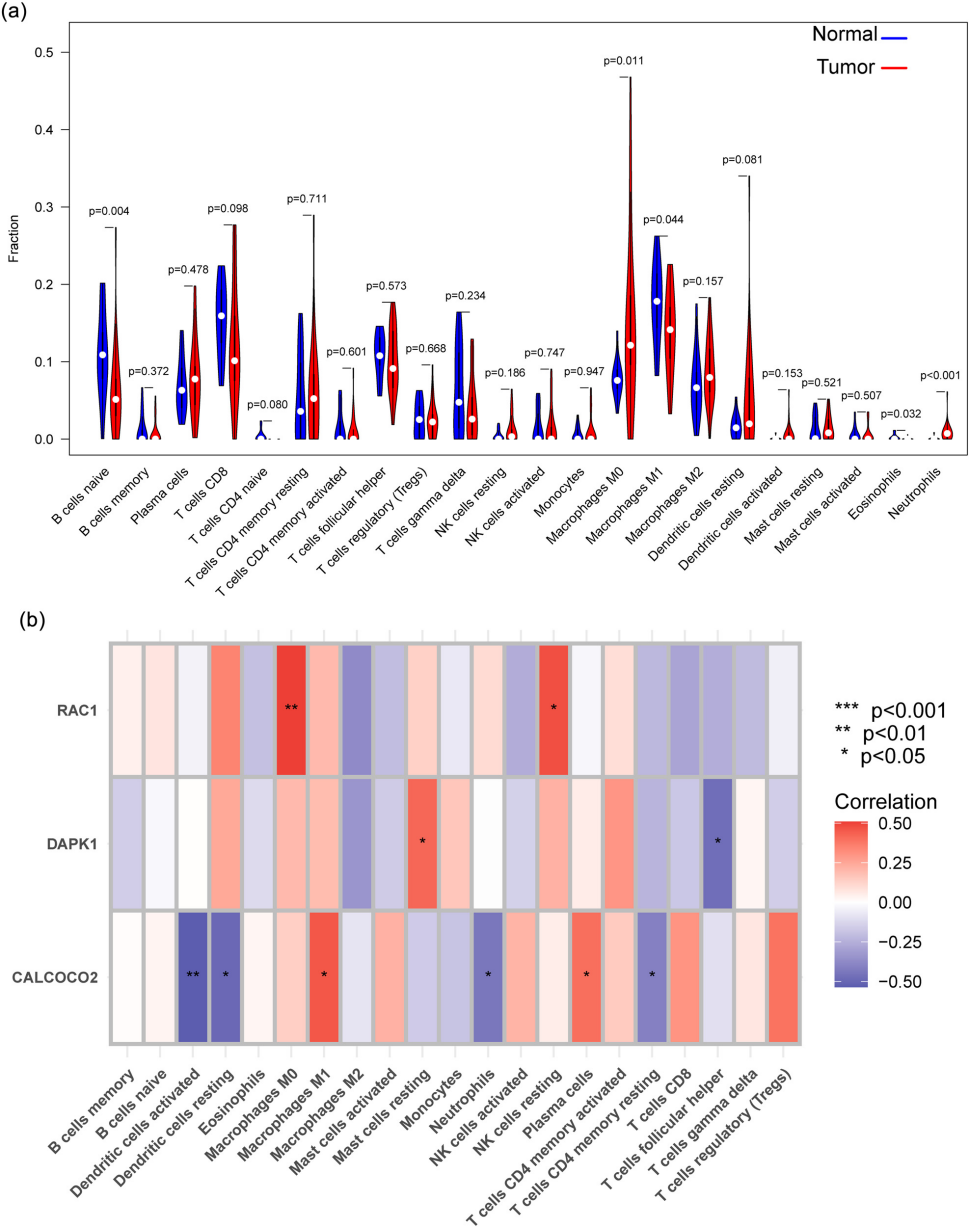
Immune infiltration analysis of CALCOCO2, DAPK1, and RAC1 in PTC. (a) The CIBERSORT algorithm. (b) Correlation between CALCOCO2, DAPK1, RAC1, and infiltrating immune cells in PTC.

Twenty-one distinct immune cell profiles were generated from PTC samples, and the fraction of tumor-infiltrating immune subsets was assessed using the CIBERSORT algorithm to further validate the association between the expression of three biomarkers and the immune microenvironment (Figure 7a). The results from the difference and correlation analyses showed that the levels of CALCOCO2 were positively associated with macrophages M1 and plasma cells, while negatively associated with dendritic cells activated, dendritic cells resting, neutrophils, and T cells CD4 memory resting. Moreover, the levels of DAPK1 were positively associated with mast cells resting, while negatively associated with T cells follicular helper. Finally, we observed that the levels of RAC1 were positively associated with macrophages M0 and NK cells resting(Figure 7b).

## 4 Discussion

PTC is one of the most common types of thyroid cancer, characterized by its slow growth and originating from the follicular cells of the thyroid gland [3]. Current advancements in treatment encompass a variety of approaches. The primary treatment method is surgical removal, which can involve partial thyroidectomy, total thyroidectomy, or lymph node dissection based on factors such as tumor size, extent, and spread [37,38]. After surgery, radioactive iodine therapy has emerged as a prevalent technique to eliminate any residual thyroid tissue or cancer cells by exploiting the uptake of radioactive iodine by PTC cells. Given the potential removal of a portion or the entire thyroid gland during surgery, patients necessitate lifelong replacement therapy with thyroid hormones to sustain optimal hormone levels in the body. Regarding prognosis, PTC typically has a favorable outlook. Prognostic factors include tumor size, patient age, gender, staging, and pathological features. In cases of early diagnosis and timely treatment, most patients experience good survival rates. However, certain high-risk factors can contribute to recurrence or metastasis. In recent years, the development of technologies like genetic testing and molecular targeted therapy has brought hope to the treatment of PTC [39,40]. Personalized treatment plans can be tailored based on a patient’s genetic characteristics and pathological features to enhance treatment effectiveness [41,42]. In summary, PTC treatment and prognosis continue to advance, offering patients a range of treatment options and improved prospects. The main diagnostic methods for PTC include fine needle aspiration biopsy, ultrasound examination, and surgical excision biopsy. However, these methods have certain limitations, including diagnostic uncertainty, overlapping features between benign and malignant characteristics, the risk of misdiagnosis and missed diagnosis, as well as challenges that could impact treatment decisions. Therefore, an accurate diagnosis of PTC requires a comprehensive consideration of various clinical information to achieve more reliable diagnostic outcomes.

Autophagy is an intracellular BP aimed at maintaining the stability and normal functioning of the cell’s internal environment. This process involves the degradation of cellular waste, damaged organelles, and proteins into basic molecules for the cell to reuse [43]. Autophagy plays a role in clearing aged, damaged, or unnecessary cellular components and is also crucial for the cell’s response to conditions such as nutrient deficiency, oxygen limitation, infection, and other stressors. By maintaining cellular equilibrium, autophagy is essential for cell survival and adaptability [44,45]. However, in certain cases, autophagy may also be associated with the development of diseases. For instance, an overabundance of autophagy has the potential to excessively degrade cellular organelles, possibly contributing to diseases such as neurodegenerative disorders. In the context of thyroid cancer, studies suggest that autophagy is implicated in the onset, progression, and development of resistance to treatment in thyroid cancer [46]. In thyroid cancer, autophagy may have a dual role, both promoting and inhibiting tumor growth, depending on specific circumstances and cellular environments. On one hand, autophagy may contribute to the survival and growth of thyroid cancer cells. In the tumor microenvironment, nutrient supply may be unstable, prompting cells to activate autophagy to acquire essential nutrients, thus supporting tumor growth and dissemination. On the other hand, some studies suggest that autophagy might play a role in inhibiting the development of thyroid cancer. By triggering autophagy in cancer cells, it is possible to diminish their energy supply and intracellular nutrients, thereby decelerating tumor growth. Moreover, autophagy may contribute to eliminating abnormal proteins and organelles, resulting in reduced cellular stress and damage, ultimately assisting in the maintenance of cellular homeostasis. In this study, we analyzed GSE33630 datasets and identified 127 DE-ARGs. The KEGG assays provided insights into the pathways associated with these DE-ARGs, revealing their involvement in critical processes such as pathways in cancer, protein processing in endoplasmic reticulum, MAPK signaling pathway, apoptosis, toll-like receptor signaling pathway, neurotrophin signaling pathway, and regulation of autophagy. This study contributes to our understanding of the intricate role that ARGs play in PTC. The identification of DE-ARGs and their functional implications sheds light on potential mechanisms underlying thyroid cancer’s pathogenesis and progression. These findings offer valuable insights into the molecular landscape of thyroid cancer and suggest potential avenues for therapeutic exploration. It was important to validate and further explore the functional significance of the identified DE-ARGs in the context of thyroid cancer through additional research and experimentation.

Machine learning plays a significant role in thyroid cancer research. By leveraging vast amounts of clinical and molecular data, it can greatly enhance the accuracy of thyroid cancer diagnosis, predict patient prognosis, guide treatment strategies, and advance the development of new drugs [47]. In the realm of early diagnosis, machine learning analyzes medical imaging data such as ultrasound, computed tomography scans, and magnetic resonance images, aiding doctors in identifying thyroid nodules’ characteristics and distinguishing between malignant tumors and benign lesions [48]. Through training models to recognize visual features of tumors, machine learning contributes to heightened accuracy in early diagnosis. Furthermore, machine learning can categorize thyroid cancer into different subtypes by analyzing its molecular features, such as gene expression and proteomics data. This enables a better understanding of the biological traits and clinical manifestations of various subtypes, supporting the formulation of more precise treatment strategies [49]. Another vital role of machine learning in thyroid cancer research lies in tailoring individualized treatment approaches. By incorporating patients’ clinical information and molecular characteristics, machine learning can predict the effectiveness of various treatment options, guiding doctors in formulating personalized treatment plans to prevent over- or under-treatment and ultimately enhance treatment outcomes for patients. Machine learning can also combine clinical, molecular, and imaging data to predict thyroid cancer patients’ survival rates and the risk of disease progression, enabling doctors to better assess patient prognosis and implement appropriate treatment and monitoring measures. In terms of drug discovery and targeted therapies, machine learning, by analyzing extensive molecular data, can identify novel drug targets associated with thyroid cancer, accelerating the development of new medications. Additionally, machine learning can predict the interactions between drug molecules and targets, aiding in the design of more effective targeted therapy drugs. Lastly, machine learning plays a role in integrating diverse data types, including clinical, molecular, and imaging data, extracting biological information from them, and revealing the underlying mechanisms and key driving factors of thyroid cancer. In summary, the multifaceted contributions of machine learning in thyroid cancer research propel scientific advancements, enhance patient treatment outcomes, and foster the development of drug therapies and personalized medicine. In this study, we performed two machine learning algorithms and identified three critical diagnostic genes, including CALCOCO2, DAPK1, and RAC1. Then, we developed a diagnostic model based on the three genes and confirmed its diagnostic value in screening PTC specimens from non-tumor specimens with AUC >0.9. Moreover, the diagnostic value of the new model was further confirmed in GSE29265 datasets and our cohort. Our findings highlighted the potential of this new model used as a novel diagnostic marker.

CALCOCO2 is a protein involved in the autophagy process. It is localized in both the cytoplasm and the cell nucleus and plays a significant role in the autophagy pathway [50]. In tumors, the role of CALCOCO2 has also attracted research attention. Some studies suggest that CALCOCO2 may have a dual role in tumor development. On one hand, the expression levels of CALCOCO2 could be modulated, thus affecting the growth and survival of tumor cells. On the other hand, as a protein closely associated with autophagy, CALCOCO2 might regulate the extent of autophagy in tumor cells under certain circumstances [51,52]. The role of autophagy in tumors can either promote the survival of tumor cells or inhibit tumor growth, depending on the type of tumor, the environment, and intracellular signaling. However, the expression and function of CALCOCO2 in PTC have not been investigated. In this study, we found that CALCOCO2 was lowly expressed in PTC specimens. Importantly, we found that CALCOCO2 may have a dual role in tumor development. On one hand, the low expression of CALCOCO2 could impact key BP such as the cell cycle, chemokine signaling pathway, cytokine-receptor interaction, natural killer (NK) cell-mediated cytotoxicity, NOD-like receptor signaling pathway, and P53 signaling pathway. These findings suggest its potential regulatory roles in tumor proliferation and immune modulation. Then, we further analyzed the association between CALCOCO2 and immune cells. Importantly, we observed that the levels CALCOCO2 were positively associated with macrophages M1 and plasma cells, while negatively associated with dendritic cells activated, dendritic cells resting, neutrophils, and T cells CD4 memory resting. This result suggests that CALCOCO2 may play an important role in immune regulation within tumors. The correlation between CALCOCO2 expression levels and various immune cell subtypes implies its potential influence on regulating the activity and function of immune cells. Specifically, the positive correlation between CALCOCO2 and macrophages M1 and plasma cells suggests a role in stimulating immune responses and promoting inflammation. Simultaneously, the negative correlation between CALCOCO2 and certain immune suppressive cell subtypes such as activated dendritic cells, resting dendritic cells, neutrophils, and CD4 memory resting T cells implies its involvement in inhibiting or modulating the functions of these inhibitory immune cells. Overall, CALCOCO2 may modulate the tumor immune environment by affecting interactions and functions of diverse immune cells.

DAPK1 (Death-Associated Protein Kinase 1) is a protein kinase that was initially discovered to be associated with the process of apoptosis (cell death). It functions as a signaling molecule within cells and plays a crucial role in various cellular processes including apoptosis, autophagy, immune regulation, and cell movement [53,54]. In the context of apoptosis, DAPK1 is considered a significant regulatory factor. It can phosphorylate downstream proteins such as structural proteins and transcription factors to mediate apoptotic signals. This positions DAPK1 within the pathways of cell death, contributing to the regulation of whether a cell undergoes apoptosis. Furthermore, DAPK1 is involved in regulating autophagy, a process through which cells degrade and recycle cellular components to maintain homeostasis [55]. The phosphorylation of proteins associated with autophagy by DAPK1 impacts the advancement of cellular autophagic processes. In the context of cancer, extensive research has been conducted on the role of DAPK1. Some studies propose that aberrant expression of DAPK1 is correlated with the initiation and progression of various tumor types. In specific instances, diminished levels of DAPK1 expression are associated with drug resistance and unfavorable prognoses in tumors. Additionally, DAPK1 can impact tumor growth and metastasis through mechanisms such as regulating apoptosis, suppressing cell migration, and inhibiting tumor angiogenesis [56,57,58]. In this study, we found the expression of DAPK1 was distinctly decreased in PTC specimens. Importantly, we observed that the down-regulation of DAPK1 may activate the chemokine signaling pathway, hematopoietic cell lineage, JAK-STAT signaling pathway, and leishmania infection signaling pathway. In the context of thyroid cancer, these activated signaling pathways might unveil the potential roles of DAPK1 in immune regulation, tumor microenvironment, and cellular behavior. This suggested that DAPK1 could have a certain impact on regulating tumor development and immune responses in thyroid cancer, highlighting its potential significance, although the precise mechanisms require further in-depth research and validation. Moreover, we confirmed that the levels of DAPK1 were positively associated with mast cells resting, while negatively associated with T cells follicular helper. Our findings suggested that DAPK1 may play a significant role in immune regulation in thyroid cancer. The correlations between its expression levels and specific immune cell subtypes revealed its potential impact within the TME. Specifically, DAPK1 showed a positive correlation with resting mast cells and a negative correlation with T cells follicular helper, implying its involvement in regulating immune responses and interactions among immune cells. These discoveries not only provided insights into the immune-regulatory function of DAPK1 in thyroid cancer but also offered novel directions for future therapeutic strategies and exploration of immunotherapeutic targets.

RAC1 is a member of the Rho family of proteins, belonging to the small GTPase family. It is involved in various BP within the cell, particularly playing a crucial role in cell motility, cell polarity, cell-matrix adhesion, and cytoskeletal remodeling [59]. RAC1 is capable of switching between active GTP-bound and inactive GDP-bound states on the cell membrane, thereby regulating multiple signaling pathways that influence cell morphology and function [60]. In tumors, the aberrant expression and activity of RAC1 have been extensively studied, particularly in the context of tumor invasion and metastasis processes. Some research findings suggest that RAC1 might be involved in regulating the migration, infiltration, and metastasis of tumor cells, subsequently impacting the invasive and metastatic capabilities of tumors. Elevated activity of RAC1 could potentially facilitate the infiltration of tumor cells, enabling them to invade surrounding tissues and blood vessels [61,62,63]. A previous study reported that the variation of RAC1 may lead to acquired resistance to dabrafenib in patients with thyroid cancer and is associated with the process of dedifferentiation. This suggests that RAC1 may play a significant role in thyroid cancer, particularly in terms of drug resistance and cellular differentiation [64]. In this study, we found that RAC1 expression was distinctly increased in PTC specimens, suggesting its oncogenic roles in PTC progression. In our research, the expression levels of RAC1 are positively correlated with macrophages M0 and resting NK cells. This suggested a potential role for RAC1 in regulating the activity and functionality of immune cells. Specifically, RAC1 may play a role in modulating the activity of immune cells within the TME, including macrophages and resting NK cells. This association could be relevant to immune evasion, immune surveillance, and responses to immunotherapy in the context of tumor progression.

There are still certain limitations in our study. First, although additional classification of PTC phenotypes is possible, we limited our analysis to tumor and non-tumor tissues. Second, the results of this study need to be verified using bigger databases and representative sample sizes. It will be important for future studies to verify the two biomarkers through *in vivo* and *in vitro* functional investigations.

## 5 Conclusion

We screened three critical diagnostic genes for PTC patients and developed a novel diagnostic model. We provided evidence that CALCOCO2, DAPK1, and RAC1 may also be involved in the regulation of the TME of PTC patients. To better understand the pathophysiology and therapy of PTC, we will keep an eye on these genes.
